# Clinical predictors of cardiac autonomic neuropathy in patients with type 1 diabetes

**DOI:** 10.1186/1758-5996-7-S1-A15

**Published:** 2015-11-11

**Authors:** Cinthia Minatel Riguetto, Caroline Rigoleto Takano, Maria Cândida Ribeiro Parisi, Elizabeth João Pavin, Arnaldo Moura Neto

**Affiliations:** 1 UNICAMP, Campinas, Brazil

## Background

Cardiac autonomic neuropathy (CAN) is frequently underdiagnosed. The prevalence of CAN rises with diabetes duration and poor glycemic control. Individuals with DM and CAN have an increased mortality risk, up to 53% five yrs. after diagnosis. Early identification can improve treatment, quality of life and mortality.

## Objective

Our aim was to determine the prevalence of CAN in patients with type 1 diabetes (T1D) and its association with clinical characteristics.

## Materials and methods

We evaluated 102 patients with T1D (67% female) divided in 2 groups: with and without CAN. Mean age and HcA1c were 34,27±10,96 yrs. and 9.0±2.0%, respectively. CAN was assessed by Poly-Spectrum software using standardized cardiovascular reflex testing and measures of heart rate variability. Statistical significance was set at 5%.

## Results

CAN was diagnosed in 39 (38.2%) patients. No statistically significant differences were found in age (34.87±9.71 vs. 33.90±11.74 yrs.; p=0.467), age at diagnosis (15.10±9.16 vs. 17.38±11.29 yrs.; p=0.495) and HbA1c (9.26%±2.04 vs. 8.84%±2.07; p=0.144) between groups. Hypertension and dyslipidemia were seen more frequently in patients with CAN (61.5 vs. 19%; p≤0.001 and 51.3 vs. 22.2%; p=0.002, respectively). Patients with CAN had higher total cholesterol (p=0.009) and triglycerides (p=0.004). Patients with CAN complained more often of post-prandial sweating and orthostatic hypotension (35.9 vs. 14.3%; p=0.011 and 51.3 vs. 30.2%; p=0.033, respectively). Other symptoms questioned were similar between groups, including hypoglycemia (p=0.7). CAN showed a rising prevalence as complication severity increased. For retinopathy, the frequency of CAN was 54.2%, 60%, 75% and 80% in those with nonproliferative, proliferative, unilateral and bilateral blindness, respectively (p<0.001). Regarding nephropathy, CAN was present in 41.2%, 75%, 88.9%, 100% and 100% in patients with microalbuminuria, macroalbuminuria, chronic kidney disease, hemodialysis and kidney transplant, respectively (p=<0.001). Diabetic neuropathy, motor sensory neuropathy/symmetric polyneuropathy and more than one neuropathy were seen in 72% and 100% of patients with CAN, respectively (p=0.001).

## Conclusions

These results support an association of increased CAN prevalence and chronic complications and their severity. CAN was also associated with hypertension and dyslipidemia, but with few autonomic symptoms (post-prandial sweating and orthostatic hypotension). As expected, HbA1c had no relevance in CAN occurrence.

**Figure 1:**
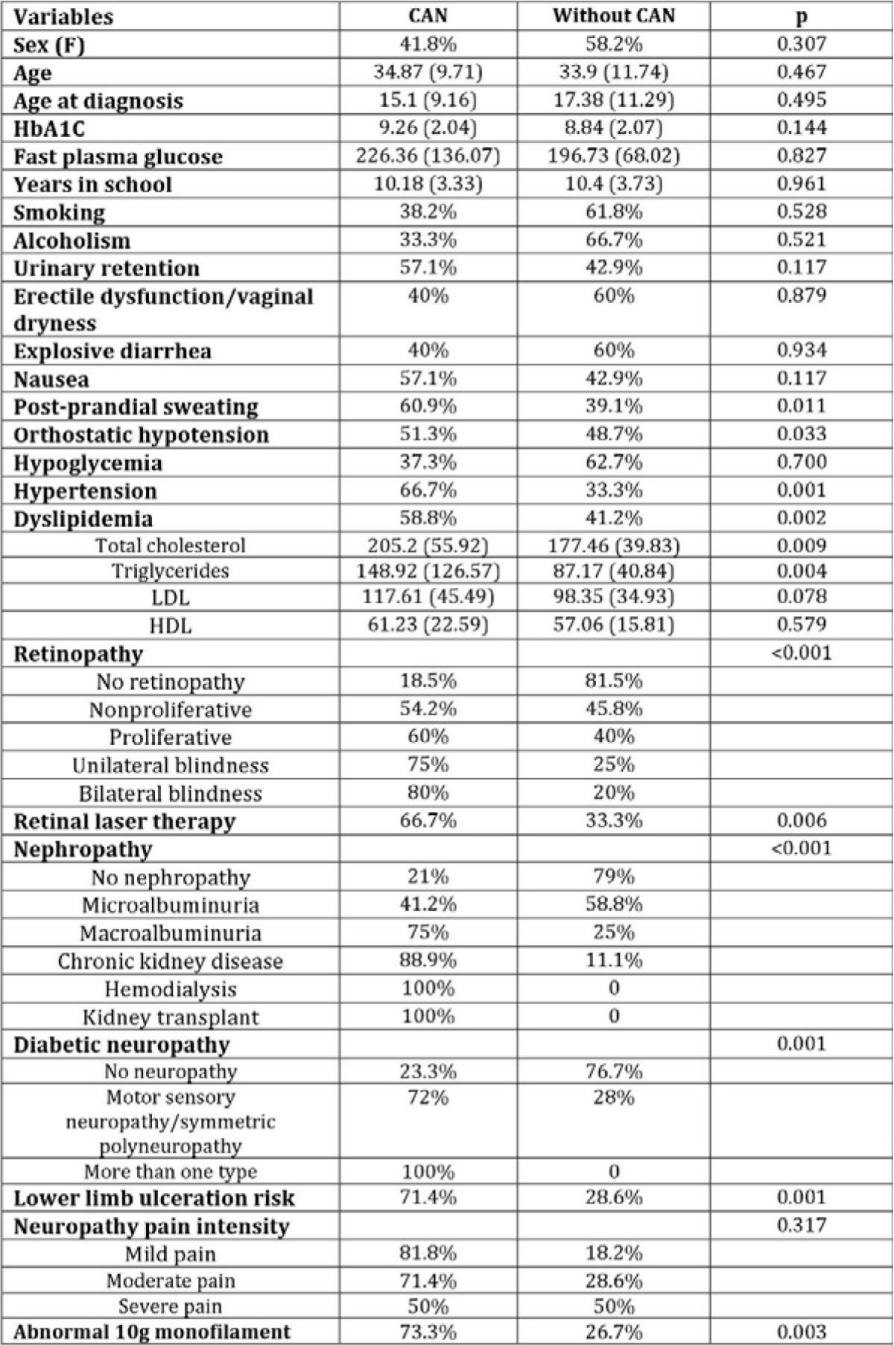
Clinical predictors of cardiac autonomic neuropathy in patients with type 1 diabetes. Values are shown as frequency (%) and mean (standard deviation).

